# Green tea improves rat sperm quality and reduced cadmium chloride damage effect in spermatogenesis cycle

**DOI:** 10.25122/jml-2018-0005

**Published:** 2018

**Authors:** Reza Mahmoudi, Arsalan Azizi, Soheila Abedini, Vahid Hemayatkhah Jahromi, Hassan Abidi, Mehrzad Jafari Barmak

**Affiliations:** 1. Cellular and Molecular Research Center, Yasuj University of Medical Sciences, Yasuj, Iran; 2. Biology Department, Azad University of Jahrom, Jahrom, Iran

**Keywords:** Cadmium chloride, Green tea, MDA, Testis, Sperm parameters

## Abstract

**Introduction:** Testicular tissue is part of the reproductive system that some mineral compounds such as cadmium chloride (CdCl_2_) destroy. Green tea (Camellia sinensis) extract can reduce the tissue damage caused by toxins due to its antioxidant properties. The aim of this study was to evaluate the effect of green tea extract on sperm quality in cadmium chloride toxicity.

**Materials and Methods:** In the present study, male Wistar rats were allotted randomly into four groups, namely control group (C), CdCl_2_ (1.5mg/kg), GT 1.5% (w/v) and in combinationCdCl_2_+GT groups. CdCl_2_ was injected intraperitoneally (1.5 mg /kg) whereas the green tea extract was administrated orally. At 13, 25 and 49 days after treatment, the rats were euthanized and the reproductive organs (testes, epididymis) were excised and used for sperm analysis and histo-morphometric examinations.

**Results:** The mean of the diameter of seminiferous tubes, the number of spermatogonia, Sertoli, Leydig cells and thickness of the germinal layer in the testis were significantly increased (P<0.05) in all groups compared to the CdCl_2_ group (P<0.05). Sperm motility, sperm count and testosterone were significantly decreased in the CdCl_2_ group compared to all groups of treatment (p<0.05). The mean of MDA was significantly increased in the CdCl_2_ group compared to other groups (p<0.05).

**Conclusion:** Green tea has an antioxidant effect that reduces the effects of free oxygen radicals produced from toxins such as cadmium chloride. In addition, it could decrease lipid peroxidation of the cell membrane and ultimately prevent the destruction of tissues in the long run.

## Introduction

Infertility affects 10–15% of couples worldwide with rates steadily increasing in industrialized areas. Males are found to only be responsible for 20–30% of infertility cases and contribute to 50% of cases overall [1, 2]. Different causes are associated with male infertility such as the quality of semen and sperm abnormalities [3]. Infertility may also occur due to genetics, nutrition, environmental pollution, and aging and chemical pollution may increase the rate of this type of disorders appearing in developing or developed countries. People are exposed to several types of environmental pollutants at different phases in their lifespan, the majority of these being harmful [4, 5].

Cadmium is an environmental pollutant that can be found in the metal plating industry, chemical fertilizers, plastic products, and dyes. Cadmium can easily contaminate soil and water then transfer to plants which is the first ring of the food chain [6, 7]. People and animals, through consumption of cadmium-contaminated agricultural products, concentrate this toxic metal in their various tissues. Cadmium chloride poisoning can lead to nephrotoxicity, osteoporosis, cardiovascular diseases, testicular necrosis, prostatic and testicular cancers, renal failure and neurodegenerative conditions [8–11]. In cadmium chloride-exposed mammals, many main organs are affected including the testes, brain, liver, and kidneys. Buha et al. reported that the most noted effect in rats given a single parenteral dose of cadmium chloride was testicular necrosis [12–14]. In addition to that, it was reported that spermatogenesis is damaged by free radical toxicity. Spermatogenesis is a cellular procedure by which a subpopulation of type A spermatogonia, namely spermatogonia stem cells, divide and differentiate into sperm [15, 16]. Spermatogonia stem cells are held in a specialized microenvironment called the niche that involves a combination of the male germ cells, somatic cells, and extracellular matrix. Sertoli cells are the main somatic cells in mammalian testis [17, 18].

The gonadotropin-releasing hormone (GRH) is secreted in the hypothalamus and, by acting on the anterior pituitary, stimulates the production and release of the LH and FSH hormones. FSH encourages sperm production in the spermatozoa and improves follicular cells growth from the ovary and LH stimulates Leydig cells in males to produce testosterone and oocyte release in females. An obstacle in the production and secretion of these hormones affects the process of spermatogenesis and folliculogenesis [17, 19].

Traditional medicine, using the properties of different herbs, can treat most diseases by using appropriate herbal compounds [20]. Many investigators proposed that one possible mechanism of cadmium chloride toxicity is the impairment of pro-oxidant and antioxidant equilibrium by the production of reactive oxygen species (ROS) [21]. Tissue levels of lipid peroxide are proven to show oxidative stress. Moreover, many investigators recorded that acute, as well as chronic cadmium chloride exposure is associated with elevated lipid peroxidation (LPO) in many organs, including male sex organs [22]. The use of herbal medicine gains ground every day and still finds an extensive use worldwide as traditional herbs have more acceptance than prescription medicine in any civilization. This was often attributed to an increased safety to that of pharmaceutical drugs from the point of view of the patient. Also, patients believe that by using this type of medication, there is no demand for a physician and it may be a relevant effort to compensate for drug failure.

Green tea (Camellia sinensis) serves as one example of traditional medication. Different studies showed that both green and black tea contain flavonoids such as quercetin, catechins and myricetin, which have strong antitoxic and anticarcinogenic effects. Research studies were done on green tea as antioxidants in it eliminate free radicals in the human body, decreasing the risk of heart disease, stroke, thrombosis of the blood vessels and blood sugar levels [22, 23].

Antioxidant compounds are one of the ingredients of green tea. All variants of green tea contain phenolic compounds which illustrate antioxidant activity [14]. Studies have shown drinking medicinal green tea daily may protect humans from different cancers such as prostate and colon cancers. It has been proven that daily green tea can also inhibit the progress of kidney and liver diseases and that it also provides lifelong protection against coronary disease [14].

The aim of this study was to evaluate the preventive effect of green tea extract on rat testicular tissues histopathology and sperm quality on rats suffering toxic effects induced by cadmium chloride.

## Materials and Methods

### Green Tea Extract

The green tea extract solution was prepared by adding 1.5 g tea to 100 ml of boiling water in a flask and stirring in a water bath shaking incubator for 10 min. The extraction solution was cooled to room temperature and then filtered through Whatman No. 1 filter paper. Chemically, green tea leaves contain a polyphenolic compound, more commonly known as Catechins. Green tea solution was provided as the patients’ sole source of drinking water until days 13, 25 and 49 according to the spermatogenesis cycle [21, 24].

### Administration of Cadmium Chloride

Cadmium chloride pure powder was obtained from Sigma-Aldrich (Germany), and distilled water was used as a solvent for the preparation of cadmium chloride administrable soluble. Then, the obtained soluble was given as a single dose injection intraperitoneally (1.5mg/ kg) in the experimental groups [12].

### Animals

Forty-eight male Wistar rats (about 180–200g body weight) were purchased from the Pasteur Institute (Tehran, Iran). Animals were acclimated in the animal facility of Yasuj University of Medical Sciences for 13, 25, 49 days, at a temperature of 24 ± 1 °C and humidity of 55 ± 5 %. The local committee on Animal Care and Use (Yasuj University of Medical Sciences) approved the experimental procedures.

### Protocol Study

Male rats were randomized into four groups (12 rats each). Control, green tea (GT), cadmium chloride (CdCl2) and cadmium plus green tea extract (CdCl_2_+GT). The control group (C) orally received sterile distilled water. The GT group (1.5% w/v) was provided a green tea solution as their sole source of drinking water daily at the end of the 13th, 25th and 49th days. The cadmium chloride group was injected intraperitoneally with cadmium chloride at a single dose (1.5mg/kg) and orally received sterile distilled water. The CdCl_2_+GT group was injected intraperitoneally cadmium chloride (1.5 mg/kg) and administrated green tea solution (1.5% w/v) until the end of the treatment. According to the spermatogenesis cycle of the rats (40–52 days), at the end of the 13th, 25th and 49th days, four rats from each group were weighed and then anesthetized by ether solution. The blood specimen was collected into dry tubes via cardiac puncture, centrifuged at 3,000 rpm for 5 minutes, and then serum was collected and kept at –10°C for further analysis. Testicles and epididymides were removed from euthanized rats and weighed.

### Sperm Quality: Sperm Count and Motility

The sperm quality outcome measurements were taken for sperm characteristics such as motility, morphology, viability and sperm count. To investigate the quality of the sperms, the caudal part of the epididymis was sliced in Hank’s buffer solution with a scalpel blade in a Petri dish. The suspension was kept at 37ºC for a minimum of 10 minutes to allow for the sperms to disperse in the medium. Six epididymides were collected from each of the rats in each experimental group. Further sample analyses included counting motile (fast and slow) and immotile sperms in a total of 500 sperm samples, and the results were expressed in a percentage. The sperm suspension was gently mixed 10 times using a measuring pipette and placed in a hemocytometer under a Zeiss microscope at a final magnification of × 400, and the total numbers of sperms were counted by multiplying by ×10^6^/mm3 [15].

### Histological Study

Testicular tissues were fixed in 10% buffer formalin, then processed by dehydrating in ascending grades of ethanol alcohols, cleared in xylol, cast, embedded, cut at 5 µm thickness by microtome and stained with hematoxylin-eosin for microscopic examination [17].

### Morphometric Analysis

Samples were measured using linear eyepiece grids and eyepiece grid reticles on the light microscope for the seminiferous tubule diameter, the height of the germinal layer, spermatogonia count, Leydig and Sertoli cell count [25].

### Testosterone Assay Procedure

An enzyme-based immunoassay (EIA) system was used to measure the testosterone level in the serum samples collected. The EIA kit from Abcam Co. (London, UK) contained a testosterone EIA enzyme label, testosterone EIA substrate reagent, and EIA quality control sample. The EIA kit used had a sensitivity of approximately 0.3nmol/M (0.1g/mL) of testosterone.

### Malondialdehyde (MDA) Measurement

The lipid peroxidation of gastric tissue was determined according to a previously described method. As the end product of the lipid peroxidation, MDA chain reacts with thiobarbituric acid (TBA) to produce a colored product (TBA-MDA) which absorbs light at 535 nm in acidic solution. Briefly, the serum samples (500 µL) were mixed with 2 mL of the reaction solution containing 100 mL HCl 0.25 M, 15 gr trichloroacetic acid (TCA) and 375 mg TBA and then heated in boiling water for 15 minutes. After cooling, MDA concentrations were determined spectrophotometrically by the absorbance of TBA reactive substances at 532 nm [4].

### Statistical Analysis

All experiments were performed in four samples for each group at the end of the 13^th^, 25^th^ and 49^th^ days (n=48), and results were expressed as a mean±standard error of means (SEM). Statistical analyses were performed by SPSS using one-way ANOVA, followed by Tukey’s post hoc test. Statistical significance is indicated by *p < 0.05, **p < 0.01, ***p < 0.001 and ****p < 0.0001.

## Results

### Effect of Cadmium Chloride on Sperm Quality

Table 1, 2 and 3 show the sperm characteristics in all four groups in days 13, 25 and 49. The means of sperm motility, sperm count and testosterone were significantly increased in the Control group compared to the CdCl_2_ group in days 13, 25 and 49 of treatment (p<0.05). The means of sperm motility, sperm count and testosterone were significantly increased in the GT group compared to the CdCl_2_ group in days 13, 25 and 49 of treatment (p<0.05). On the other hand, administration of GT to healthy animals did not cause a significant effect on relative sperm quality in comparison to the control and GT plus CdCl_2_ groups. In fact, cadmium chloride significantly reduced relative sperm quality and sperm count of testis compared to the control and GT groups (p<0.05). The means of sperm motility, sperm count and testosterone were significantly increased in the GT plus CdCl_2_ group compared to the CdCl_2_ group in days 13, 25 and 49 of treatment (p<0.05). Also, the mean of the sperm count was significantly decreased in the GT plus CdCl_2_ group compared to the Control and GT groups on the 13^th^ day of treatment. Analysis of rats belonging to the GT plus CdCl_2_ group resulted in significant relative sperm quality increase compared to the cadmium chloride group. This study showed that the Catechins antioxidant in green tea leaves has been able to reduce the destructive effects of CdCl_2_ over a short period of time. The mean of sperm morphology and body weight were not significant in the CdCl_2_ group compared to other groups in days 13, 25 and 49.

**Table 1:** Effects of cadmium chloride, green tea on sperm quality and testes tissue on the 13^th^ day

Groups		CdCl_2_+GT	CdCl_2_	GT	Control
Sperm motility%	Fast	10.57±3.52	2.23±2.08^a^	11.67±2.88	12.67±1.88
	Slow	35.14±6.51	8.63±6.91^a^	38±5.92	46±4.52
	Non-motile	49.29±6.58	89.14±6.37^a^	53.33±6.87	63.33±5.66
Sperm morphology%	Normal	95.86±0.41	96±0.45	97.33±0.32	97±0.43
	Abnormal	3.14±0.41	3±0.48	2.87±0.01	2.7±0.62
Sperm counting (N/mm^3^)		113.23±7.72^b^	67.85±9.42^a^	168.67±7.86	173±8.86
testosterone (ng/dl)		2.31±1.31	1.83±1.25^a^	5.38±1.24	5.65±1.26
Weight (gr)		278.14±10.16	267.17±7.95	286.67±8.33	283.33±9.33
**Histological change**					
Diameter of seminiferous tubule (μ)		274.56±15.74^a^	248.57±10.43^a^	304.68±14.67	314.25±18.23
Germinal layer thickness (μ)		58.74±4.31^b^	38.18±4.92^a^	90.78±6.84	91.27±9.11
Number of spermatogonia (mm^2^)		1063.27±29.14^a^	1031.13±23.50^a^	1290.68±38.12	1286.23±45.91
Number of leydig cells (mm^2^)		563.21±36.37^a^	408.16±25.11^a^	649.53±30.41	668.34±26.32
Number of sertoli cells (mm^2^)		102.45±16.32^a^	84.52±15.21^a^	134.12±28.16	136.50±15.21

Sperm motility, sperm count and histological change of testes showed a significant increase in the control group compared to CdCl_2_ GT and GT plus CdCl_2_. Results are represented as Mean±SEM. Statistical significance is indicated; ^a-b^ Mean in the same column carrying different superscript letters are significantly different (p<0.05). Data for each time point was repeated for n= 6 times.

**Table 2:** Effects of cadmium chloride, green tea on sperm quality and testes tissue on the 25^th^ day

Groups		CdCl_2_+GT	CdCl_2_	GT	Control
Sperm motility%	Fast	17.33±2.86	5.33±2.21^a^	18.33±1.85	19±3.06
	Slow	30.67±4.33	10.67±3.82^a^	33±4.53	36±3.78
	Non-motile	50±6.11	84±7.62^a^	54.67±11.39	50±6.56
Sperm morphology%	Normal	97±0.01	97±0.01	97±0.01	96.67±0.33
	Abnormal	3±0.24	3±0.15	3±0.08	3.33±0.33
Sperm counting (N/mm^3^)		143.42±6.36	128.33±8.06^a^	165.67±6.17	176.67±7.31
testosterone (ng/dl)		3.87±1.32	1.58±1.06^a^	5.86±1.86	6±1.06
Weight (gr)		258.33±9.33	247.67±13.82	268.67±8.09	276.34±16.17
**Histological change**					
Diameter of seminiferous tubule (μ)		289.14±11.49	207.11±18.79^a^	315.14±17.87	324.76±10.48
Germinal layer thickness (μ)		87.01±9.51	38.97±6.65^a^	97.24±6.73	99.67±6.92
Number of spermatogonia (mm^2^)		1353.87±39.93	1175.45±41.24^a^	1396.86±30.63	1410.26±34.46
Number of leydig cells (mm^2^)		658.46±11.23	490.62±17.93^a^	673.17±12.91	688.54±10.52
Number of sertoli cells (mm^2^)		140.57±5.65	97±5.32^a^	143.27±4.22	148.23±9.03

Sperm motility, sperm count and histological change of testes showed a significant increase in the control group compared to CdCl_2_ GT and GT plus CdCl_2_. Results are represented as Mean±SEM. Statistical significance is indicated; ^a-b^ Mean in the same column carrying different superscript letters are significantly different (p<0.05). Data for each time point was repeated for n= 6 times.

**Table 3:** Effects of cadmium chloride, green tea on sperm quality and testes tissue on the 49^th^ day

Groups		CdCl_2_+GT	CdCl_2_	GT	Control
Sperm motility%	Fast	10±2.73	8.33±1.26^a^	13.33±2.67	13.67±1.78
	Slow	32.67±3.51	21±5.69	33.34±5.21	38.67±4.33
	Non-motile	57.33±4.04	68.67±2.85	56.33±5.01	47.66±3.84
Sperm morphology%	Normal	97±0.01	93.33±0.33	97±0.03	97±0.01
	Abnormal	3±0.01	6.67±2.33	3±0.01	3±0.33
Sperm counting (N/mm^3^)		163.33±17.85	144.67±8.76^a^	170.67±12.99	172±14.16
testosterone (ng/dl)		4.9±1.13	2.46±1.85^a^	3.56±1.76	4.66±2.06
Weight (gr)		279.33±14.33	270±21.2	300.67±13.28	283.5±19.5
**Histological change**					
Diameter of seminiferous tubule (μ)		303.11±12.88	265.33±9.97^a^	337.5±15.97	359.6±10.73
Germinal layer thickness (μ)		89.42±6.48	60.17±4.97^a^	94.06±7.19	113.13±8.52
Number of spermatogonia (mm^2^)		1393.83±29.52	1178.62±16.63^a^	1403.36±23.13	1421.42±14.36
Number of leydig cells (mm^2^)		650.51±13.32	507.57±10.17^a^	663.61±17.31	686.65±14.4
Number of sertoli cells (mm^2^)		123.43±7.27	109.56±5.28^a^	134.29±6.26	137.45±7.6

Sperm quality and histological change were not significant in the control group compared to CdCl_2_ GT and GT plus CdCl_2_. Results are represented as Mean±SEM. Statistical significance is indicated; ^a-b^ Mean in the same column carrying different superscript letters are significantly different (p<0.05). Data for each time point was repeated for n= 6 times.

### Effect of Cadmium Chloride on Histopathological Changes

Table 1, 2 and 3 show means of variable histological changes in all four groups on days 13, 25 and 49. The results also showed increases in the mean of the diameter of seminiferous tubules, germinal layer thickness, number of spermatogonia, Sertoli and Leydig cells of the rats in the control group compared to the CdCl_2_ group on days 13, 25 and 49 (P<0.05). Also, the mean values of the diameter of seminiferous tubules, germinal layer thickness, number of spermatogonia, Sertoli and Leydig cells of the rats were shown in the GT and GT plus CdCl_2_ groups compared to the CdCl_2_ group on days 13, 25 and 49 (P<0.05). Pathological changes were not significant in the control group compared to the GT and GT plus CdCl_2_ groups. The study of the histological sections showed that the germinal epithelium atrophic changes in the CdCl_2_ group. Germinal epithelium had been detached from the basement membrane and the progenitor cells dramatically damaged. In fact, cadmium chloride caused a reduction in the germinal layer’s thickness, ultimately leading to a decrease in the diameter of the seminiferous tubule. However, the cell population recovered in the rats treated in the GT plus CdCl2 group compared to the CdCl_2_ group (Fig. 2).

**Figure 1:**
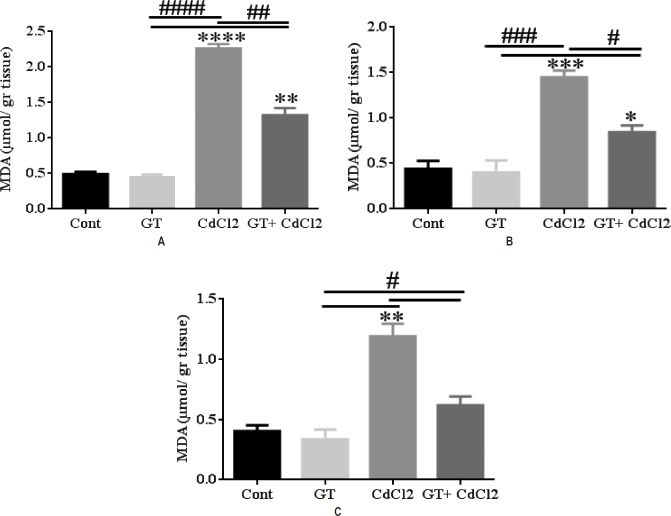
**MDA level in testes tissue.** MDA at (**A**) 13^th^, (**B**) 25^th^, and (**C**) 49^th^. Results showed a significant increase in the MDA 13, 25 and 49 days in the CdCl_2_ compared to Cont., GT and GT plus CdCl_2_. CdCl_2_ increased lipid peroxidation compared to other groups. Results are represented as Mean±SEM. Statistical significance is indicated by **p < 0.01, ****p < 0.0001, #p < 0.05, ##p < 0.01, and ####p < 0.0001. *corresponds to the comparisons between each group versus the control group. # corresponds to the internal comparisons within the various groups. Data for each time point was repeated for n= 6 times.

**Figure 2:**
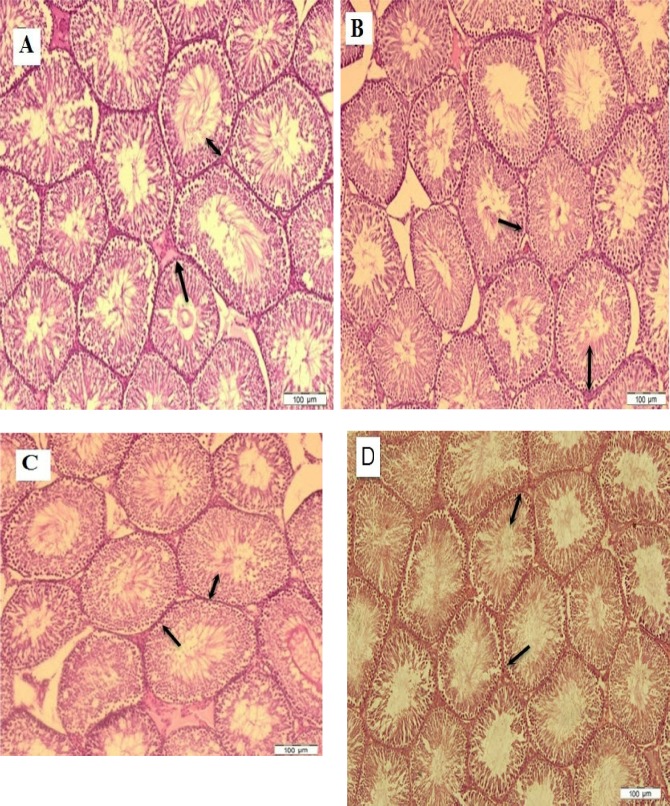

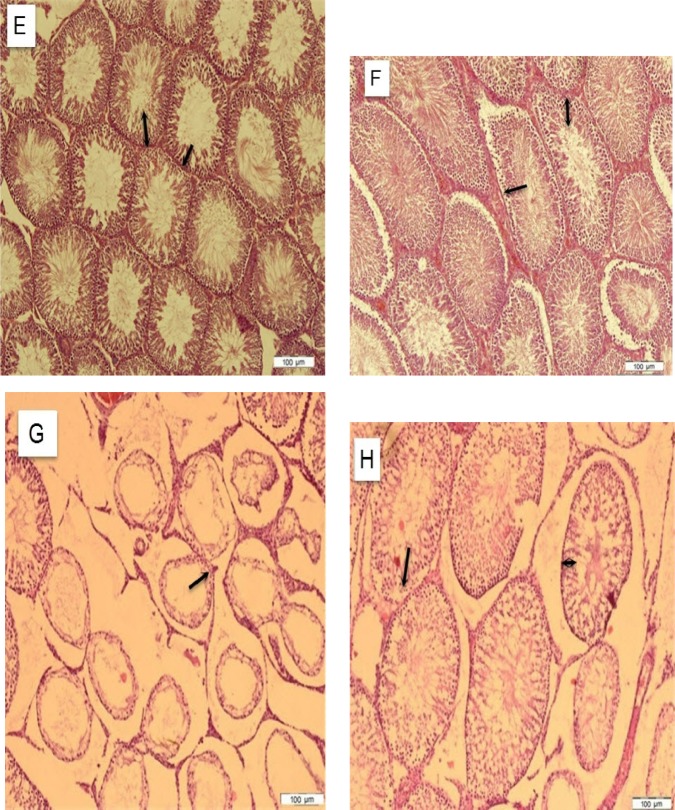
**Photomicrograph of the testicles’ histology in study groups.** In the control 13^th^ day (A), control 25^th^ (B), control 49^th^ days (C), GT 13^th^ day (D), GT 25^th^ day (E), GT 49^th^ day (F), CdCl_2_ 13^th^ day (G), CdCl_2_ 25^th^ day (H), CdCl_2_ 49^th^ day (I), GT plus CdCl_2_ 13^th^ day (J), GT plus CdCl_2_ 25^th^ day (K), GT plus CdCl_2_ 49^th^ day (L). In the animals treated with GT (D, E, F) the tubules and spermatogonia cells maintained normal form. In the animals that received cadmium chloride (G, H, I), the tubules seemed atrophic, the edema increased, germinal layer height decreased (Double side black arrow), and many spermatogonia cells (One side black arrow) were lost. No changes were observed in the CdCl2 plus GT 25 and 49 days (B) and green tea 13, 25 and 49 days (E). The antioxidant effects of green tea considerably restored the tubules and epithelial cells. (Magnificent X40, Hematoxylin-Eosin stain).

### Biochemical result

The level of MDA in tissue homogenates of testes was significantly raised in the CdCl_2_ group compared to other groups. In the GT plus CdCl_2_ group, the levels of MDA in the studied tissues were significantly reduced compared to the CdCl_2_ group on days 13, 25 and 49. Also, in the GT plus CdCl_2_ group, the levels of MDA in the studied tissues were significantly increased compared to the control and GT groups on days 13, 25 and 49 (Fig.1–3). In fact, cadmium chloride increased lipid peroxidation of the cell membrane.

## Discussion

In the present study, the preventive effect of green tea extract was investigated in an assay for histological change, exposing subjects to cadmium chloride in the spermatogenesis cycle. Spermatogenesis is an essential period of the human reproductive system. Primary sexual cells are transmitted throughout the fetal period to the seminal tubules, which initiate their normal growth and reproduction after birth and at the onset of puberty [19]. The most common cause of infertility is the disruption of sperm production and performance during the spermatogenesis cycle [16]. One of the most important causes of infertility is ROS [5, 17]. Free radicals destroy the phospholipids in the membrane of testosterone-secreting cells and reduce hormonal effects, and thus increase the incidence of infertility [3]. The toxic and destructive effects of cadmium chloride are owed to the production of ROS, peroxidation of the cell membrane and thereby the destruction of tissue cells [10, 26].

Cadmium chloride is widely distributed in the environment because of its many industrial applications. Research has shown that cadmium is one of the most important environmental and industrial pollutants. Previously, Eleawa (2014) reported that single-dose (19) Cd administration increased lipid peroxidation and increased MDA in the plasma. The present study displayed that the levels of MDA in the tissues homogenates of testes were significantly increased in the CdCl_2_ group compared with controls on the 13^th^, 25^th^ and 49^th^ days.

Cadmium chloride can decrease the growth and proliferation of testicular tissue by disrupting the pituitary-gonadal axis [1, 9, 15]. This study showed that cadmium chloride could increase a lot of ROS, thereby the spermatogonia, Sertoli and Leydig cells were destroyed. Also, increasing the death of these cells caused a reduction in the thickness of the germinal layer in the seminiferous tubule in the CdCl_2_ group compared with controls on the 13^th^, 25^th^ and 49^th^ days.

Moreover, results have shown that cadmium can cause edema and necrosis of testicular tissues, which is consistent with other researchers’ studies [27]. Sertoli cells have important effects on the control of proliferation and differentiation spermatogonia. Any disruption and destruction in this process can halt the spermatozoa generation at different levels of spermatogenesis [11, 18]. Sperm tube atrophy and spermatogonia cell decline are symptoms of sperm production disorders [1].

Various mechanisms have been proposed to explain the mechanism of cadmium chloride damage to tissue cells, which include increasing reactive oxygen species of lipid peroxidase and damaging the DNA, disrupting the antioxidant system, or defecting the apoptotic gene expression [28]. Also, one of these mechanisms explains that cadmium chloride increases the thickness of the basement membrane and reduces the production of sperm. As a result, blood flow to the somatic and sexual cells is cut off, and so it leads to a decrease in the number of spermatogonia and Sertoli cells [13]. This study assessed the Catechins antioxidant effects of green tea as a source for neutralizing lipid peroxidation and improving infertility in men.

The current study revealed that the means of sperm motility, sperm count and testosterone were significantly increased in the GT plus CdCl_2_ group compared to the CdCl_2_ group on days 13, 25 and 49 of treatment. The results of this study showed that rats injected with cadmium chloride that consumed green tea extract had less gonadal changes than the cadmium chloride group. It can be argued that oxidants’ effect is reduced by green tea and gonadal tissue damage is prevented for more extended periods.

Green tea with an antioxidant effect can reduce blood glucose, and it can be used to control diabetes, infertility, and cardiovascular disease [29]. Also, research has shown that vitamins and flavonoids and the resulting Catechins [21, 22] as a major phenolic agent in green tea play the role of antioxidants. One of the types of green tea vitamins is vitamin E, which is a potent antioxidant [9]. That supporting role has been reported in the quality and quantity of sperm, fertilization, and fertility in humans. Some authors claim that Catechins are the main factor behind the inhibition of the absorption of cadmium chloride in rats. The results showed the highest efficiency in reducing the absorption of cadmium chloride in subjects drinking green tea [14, 20].

The findings of the present study revealed that even inducing lower doses of cadmium chloride for a short period led to toxic effects. The reduction in spermatozoa parameters is the consequence of the reduction in germinal epithelial cells after exposure to cadmium chloride.

## Conclusions

The results of the present study revealed that green tea ameliorated sperm defects in cadmium chloride-induced rats and the protection mechanism of green tea reduces the effects of reactive oxygen species. For this reason, catechins existing in green tea leaves decreased cadmium chloride effects. However, this preliminary study opens a new platform for studying green tea as a potential therapeutic agent in treating reproductive complications.

## Acknowledgments

The authors are grateful to the Cellular and Molecular Research Center of Yasuj University of Medical Sciences for financial support.

## Conflict of Interest

The authors confirm that there are no conflicts of interest.
